# Engaging community stakeholders to develop a Dementia Risk Reduction Protocol for *primary care settings assisting underserved mid‐aged and older adults*

**DOI:** 10.1002/alz.087119

**Published:** 2025-01-09

**Authors:** Juliana Nery Souza‐Talarico, Sato Ashida, Larry Newman

**Affiliations:** ^1^ University of Iowa, Iowa, IA USA; ^2^ University of Iowa College of Public Health, Iowa City, IA USA; ^3^ University of Iowa College of Nursing, Iowa City, IA USA

## Abstract

**Background:**

Reducing dementia risk factors at mid‐life by 10‐25% can prevent an estimated 1‐3 million dementia cases worldwide. However, implementation of dementia risk reduction (DRR) is limited, particularly in underserved communities. Barriers include lack of awareness and access to appropriate services. Engaging community stakeholders is crucial for adapting and incorporating interventions that are feasible, acceptable, and sustainable into community care. We aimed to involve local stakeholders to develop a DRR protocol suitable for primary prevention in underserved communities.

**Methods:**

Collaborating with a university‐affiliated Mobile Clinic (U‐MC) offering free health services, we reached out to middle‐aged and older adults in two underserved communities. A Stakeholder Advisory Board (SAB) was formed, comprising eight individuals, including community members, primary care providers/students, researchers, and aging organization representatives. Relying on the recommendations of the *UsAgainstAlzheimer’s* risk reduction workgroup, the SAB employed the ACTT framework (Actions, Context, Target, and Time) to delineate DRR strategies.

**Results:**

Over five bi‐weekly meetings, the SAB designed an action plan for dementia risk screening (11 risk factors), education and follow‐up (**A**ctions), involving students and primary care professionals (**A**ctors) in community health assessments (**C**ontext) for individuals over 40 (**T**arget). Culturally‐sensitive educational materials with local resources were designed to support detected risks, with annual monitoring (**T**ime). Insights into the SAB operations like group dynamics and partnership efficacy were also evaluated to optimize collaborative efforts in healthcare delivery.

**Conclusion:**

The active collaboration with community stakeholders facilitated the development of a tailored, evidence‐based DRR protocol, reinforcing the importance of local input in creating feasible and sustainable health interventions in underserved communities. This customized approach shows promise for wider adoption in similar communities, aiming to promote primary prevention and substantially decrease the incidence of dementia.

